# Exploring the interplay between pain processing mechanisms, psychosocial factors, and functional outcomes in patients with chronic low back pain: an exploratory study

**DOI:** 10.1080/07853890.2025.2536203

**Published:** 2025-07-25

**Authors:** Jaime Jordán-López, María D. Arguisuelas, Julio Doménech, M. Lourdes Peñalver-Barrios, Marta Miragall, Rocío Herrero, Rosa M. Baños, Juan J. Amer-Cuenca, Juan F. Lisón

**Affiliations:** a Department of Biomedical Sciences, School of Health Sciences, Universidad Cardenal Herrera-CEU, CEU Universities, Valencia, Spain; b Department of Physiotherapy, School of Health Sciences, Universidad Cardenal Herrera-CEU, CEU Universities, Valencia, Spain; c Department of Orthopaedic Surgery and Traumatology, Clínica Universidad de Navarra, Pamplona, Spain; d Department of Physical Medicine and Rehabilitation, Hospital Arnau de Vilanova, Valencia, Spain; e CIBER of Physiopathology of Obesity and Nutrition (CIBEROBN), Instituto de Salud Carlos III, Madrid, Spain; f Department of Personality, Evaluation, and Psychological Treatments, University of Valencia, Valencia, Spain; g Department of Psychology and Sociology, Universidad de Zaragoza, Teruel, Spain; h Polibienestar Research Institute, University of Valencia, Valencia

**Keywords:** Chronic low back pain, biopsychosocial model, central sensitisation, quantitative sensory testing, psychological factors, functional capacity, correlation analyses and stepwise regression analyses

## Abstract

**Background:**

Chronic low back pain (CLBP) is a prevalent condition associated with disability and increased health service usage. Understanding the interrelationship between central pain processing mechanisms, psychological factors, and functional outcomes in patients with CLBP may enhance their clinical assessment and treatment. This present study aimed to explore correlations between pain intensity and pain processing mechanisms (quantitative sensory testing [QST]), psychological factors (kinesiophobia, catastrophising, and anxiety), and functional outcomes (lumbar flexion-evoked pain thresholds and functional capacity) in individuals with CLBP.

**Methods:**

In this exploratory study we recruited 50 patients with CLBP from a tertiary hospital in Valencia, Spain. Pain processing mechanisms were assessed using QST parameters (pressure pain threshold [PPT], temporal summation [TS], and conditioned pain modulation [CPM]). Psychological factors were measured through validated scales and functional outcomes were assessed *via* lumbar flexion-evoked pain threshold and the 1-minute sit-to-stand (STS60) test. Pearson correlation and stepwise regression analyses were used to examine associations and predictive relationships.

**Results:**

Pain intensity was significantly associated with reduced PPT, low CPM, slower STS60 performance, and elevated kinesiophobia, catastrophising, and anxiety levels (*p* < 0.05). Regression analysis identified the PPT and STS60 results as significant predictors of pain intensity (AdjR^2^ = 0.397, *p* < 0.001), accounting for 39.7% of the variation in lumbar pain. In turn, STS60 performance and catastrophising were significant predictors of kinesiophobia (AdjR^2^ = 0.291, *p* < 0.001) accounting for 29.1% of its variation.

**Conclusion:**

Pain intensity in patients with CLBP correlates with central sensitisation, psychological distress, and functional limitations. PPT and STS60 may serve as valuable clinical indicators of pain severity and functional impairment, respectively. These findings support a multidimensional assessment framework for CLBP, integrating sensory, psychological, and functional factors to inform comprehensive treatment strategies.

## Introduction

Chronic low back pain (CLBP) is one of the leading causes of disability worldwide and significantly affects healthcare services [1]. CLBP is a prevalent musculoskeletal disorder affecting approximately 90% of individuals at least once in their lifetime [2]. Cases categorised as non-specific CLBP lack a clearly identifiable origin, with this non-specific nature arising from the complex interplay of various factors contributing to CLBP. These factors include biophysical characteristics, neurophysiological elements such as central nervous system (CNS) modulation, and psychological elements such as kinesiophobia, catastrophising or anxiety, social determinants, lifestyle factors, occupational elements, and comorbidities [3–6].

Within the biopsychosocial framework for understanding chronic pain, several more specific mechanism-oriented models have been developed to describe the factors that may influence pain [7]. Perhaps the most well-known and influential of these is the Fear-Avoidance Model [7–9]. This cognitive-behavioural theoretical model of chronic pain posits that individuals avoid or cease performing activities or movements that previously caused them injury or pain, or that they believe may cause it [7–9]. This model explains the development and persistence of disabling CLBP in a subgroup of patients. The model suggests that pain-related disability arises from a cyclical and interactive sequence of cognitive (e.g. kinesiophobia and catastrophising), affective, and behavioural processes (such as inactivity) driven by fear [7–9]. Pain-related fear or kinesiophobia is associated with musculoskeletal pain and is considered a precursor to chronic pain, inactivity, and disability [7–9].

Over the past decade, there has been a substantial increase in research not only investigating the Fear-Avoidance Model but also exploring the mechanisms that promote and inhibit nociception in individuals experiencing chronic pain. Thus, the profound interconnection and influence between the CNS and mechanisms that underlie pain processing in cases of CLBP has been studied [5]. However, these central mechanisms of CLBP remain incompletely understood [10]. Nociceptive signals from peripheral tissues occur in parallel with maladaptive processing within the CNS, contributing to the persistence and severity of CLBP and to central sensitisation (CS) [11].

CS is defined as increased responsiveness of spinal nociceptive neurons, where their cortical projections can outlast tissue-based input or be sustained by a normally subthreshold tissue-based input [12]. Consequently, in the presence of CS, central neurons display heightened responsiveness to peripheral nociceptive input [13]. This process of central pain facilitation may result from heightened connectivity between sensory and emotional control regions in the brain as well as reduced connectivity with descending inhibitory pathways [14]. Alterations in central neuronal processing may underlie several problems such as kinesiophobia, catastrophising, anxiety, or fatigue, each of which adversely affects the pain experience [10].

Quantitative sensory testing (QST) is used to measure some mechanisms of pain. QST is an umbrella term for non-invasive psychophysical tissue-stimulation tests that provide information about altered sensory processing such as sensitivity and dysregulation of pro-nociceptive and anti-nociceptive pathways, thereby providing insights into pain mechanisms [15]. These aspects of central pain processing are assessed by the QST modalities: pain pressure detection thresholds (PPTs), which are commonly used to assess pain sensitivity, temporal summation (TS), utilised to evaluate nociceptive facilitation, and conditioned pain modulation (CPM), employed to assess descending inhibitory pathways [10,16–18].

Previous narrative reviews have reported differences between people with CLBP and healthy controls in terms of several QST metrics. Higher PPTs at remote body parts from pain, facilitated TS, and low CPM were interpreted as signs of CS and were present in individuals with CLBP [19–22]. However, the relationship between PPT, TS, and CPM with in cases of chronic pain and its associated psychological consequences such as kinesiophobia, catastrophising, and anxiety, are still not completely understood [23]

Moreover, people with CLBP often experience pain during functional activities which, in turn, reduces their functional capacity [24]. Functional capacity is widely recognised as a key indicator of individual overall health status and serves as an important predictor of morbidity and mortality [25]. A variety of functional capacity tests have been developed, including the standing long jump test, isokinetic dynamometry, the 6-minute walk test, cardiopulmonary exercise testing on a treadmill or stationary bike, or the 20-metre multistage fitness test [25]. One increasingly utilised functional capacity test is the 1-Minute Sit-to-Stand (STS60) test, which offers an attractive alternative to other assessments of overall functional capacity because of its simplicity, speed, and adaptability to use in small spaces. It is widely employed in clinical practice because of its ease of administration and its effectiveness in reflecting overall functional status [25–27].

Activity-related pain metrics such as movement-evoked pain thresholds have been used to capture information on distinct aspects of activity-related pain [24]. Furthermore, recent studies in patients with knee osteoarthritis have confirmed that movement-evoked pain shows predictive associations with important outcomes of pain, thereby enhancing the understanding of various factors contributing to the pain experience [28,29]. Therefore, emerging research is increasingly focused on elucidating the role of pain sensitivity, pain modulatory mechanisms, and movement-evoked pain thresholds in the genesis, influence, or modulation of CLBP with the aim of understanding chronic pain mechanisms and improving clinical effectiveness [6,10, 23,30,31].

These variables have only been studied collectively in knee osteoarthritis. However, while they have been examined individually in patients with CLBP, these studies lacked a more holistic perspective. To the best of our knowledge, no research to date has investigated the correlations between pain intensity, pain processing mechanisms in conjunction with various psychosocial factors, and functional outcomes in CLBP. Thus, the primary objective of this exploratory study was to investigate potential correlations between pain intensity and pain processing mechanisms (QST parameters [PPT, TS, and CPM]), psychological factors (kinesiophobia, catastrophising, and anxiety), and functional outcomes (lumbar movement-evoked pain thresholds and functional capacity) in individuals with CLBP. We hypothesised that higher levels of pain intensity are associated with CS QST indices, poorer psychological factor outcomes, and lower lumbar movement-evoked pain thresholds and functional capacity in individuals with CLBP. A secondary aim was to explore which variables were most strongly associated with pain intensity and kinesiophobia, a key component of the Fear-Avoidance Model given its recognised impact on pain-related disability and behavioural avoidance in individuals with CLBP, with the goal of identifying potential predictors for future hypothesis-driven research.

## Methods

### Study design

This observational study recruited participants from the Orthopaedic Surgery Service and Physical Medicine and Rehabilitation Service at Arnau de Vilanova Hospital in Valencia, Spain. The study was approved by the Ethics Committee at the Arnau de Vilanova Hospital in Valencia, Spain, (reference: CEIm:30/2021) as well as by the CEU Cardenal Herrera University in Valencia, Spain, (reference: CEEI21/203) in accordance with the fundamental principles established in the Declaration of Helsinki. Prior to commencing the study, all the participants received an information letter and provided their written consent to participation. Additionally, this study was conducted in compliance with the STROBE reporting guidelines for observational studies [32].

### Participants

Fifty patients diagnosed with non-specific CLBP, according to the COST B13 European guideline, [33] were enrolled in this observational study. The inclusion criteria were as follows: (1) age between 18 and 65 years; and (2) an average pain score of 3 or higher on the Pain Numerical Rating Scale (PNRS; 0 indicating no pain, and 10 indicating the worst pain imaginable) [34] in the 6 months prior, in line with the IMMPACT recommendations aimed at minimising the potential influence of individual natural history on pain [35]. Patients with comorbidities such as (1) a spinal tumour, infection, or fracture; (2) lower extremity musculoskeletal injuries (e.g. sciatica or radiating lower extremity pain, numbness, or weakness symptoms); (3) cauda equina syndrome; (4) fibromyalgia; (5) previous spinal surgery; or (6) systemic disease, were excluded. An upper age limit of 65 years was applied to minimise age-related confounding factors such as sensory decline or cognitive variability that could affect QST measures or questionnaire interpretation.

In this observational study, the sample size was calculated using G*Power 3 software [36]. The sample size was determined based on expected effect sizes reported in previous research (*d* = 0.35), ensuring adequate power to detect associations among the variables. With an alpha of 0.05 and a power of 0.80, the need for a final sample size of 46 patients was established. To account for potential dropouts, we increased the sample size by 10% to a total of 50 patients.

### Procedure

Participants completed all the questionnaires related to pain and psychological factors online. Subsequently, patients attended the physiotherapy department at Arnau de Vilanova Hospital, where the same physical therapist evaluated their pain processing mechanisms and functional outcomes. The outcomes measured in this study included pain (pain intensity), pain processing mechanisms (QST parameters [PPT, TS, and CPM]), psychological factors (kinesiophobia, catastrophising, and anxiety), and functional outcomes (lumbar movement-evoked pain thresholds and functional capacity).

#### Pain and pain processing mechanisms

1. The pain severity perceived by participants was measured using the Numerical Pain Rating Scale (NPRS-11) asking participants to rate their average low back pain over the past week. Its validity and reliability has been well-documented. The NPRS-11 is a Likert-type scale ranging from 0 (no pain) to 10 (worst imaginable pain), where the patient verbally selects the value that best describes their perceived pain intensity [34,37].
2. QST: PPT, TS, and CPM are indices of central nociceptive processing. First, baseline PPTs were assessed using algometry on the index finger (dorsal aspect of the distal phalanx) and 5 cm to the right of the spinous process of L3. The PPT is defined as the lowest pressure that, under standardised testing conditions, elicits the first sensation of pain. It is a reliable and widely used metric for assessing pain sensitivity [17,38]. The PPTs were measured using an analogue Wagner algometer (Wagner Instruments, Greenwich, CT, USA) with a probe tip surface area of 1 cm^2^, applying the algometer probe at a constant rate of 1 kg/cm^2^/s. The mean of three consecutive measurements at the index finger, taken at 30-second intervals, was used as the test stimulus in both TS and CPM protocols but was not included as a separate variable in the correlation analysis with pain severity [17].

To avoid carryover effects, TS and CPM were measured two minutes after the PPT measurements to evaluate nociceptive facilitation and descending inhibition, respectively. The degree of TS, or wind-up, was assessed in response to 10 pulses of the algometer, with an approximate pressure increase rate of 2 kg/s, at the previously defined index finger PPT. Participants were asked to use the NPRS-11 to intuitively rate the severity and discomfort of the pain associated with the first and tenth pulses. The degree of TS, reflecting the level of pain facilitation, was calculated as the difference between the final and the first PPT values during a series of three consecutive stimuli applied to the index finger region. A positive TS value indicates increased pain facilitation over repeated stimulation [17,39,40].

Following a five-minute interval, CPM was assessed by replicating the TS evaluation in association with a conditioning stimulus used to induce CPM. To create said stimulus, an occlusion cuff was applied to the left arm and inflated at a rate of 20 mmHg/s until the patient reported the ‘first sensation of pain.’ The pressure reached at this point was then maintained for 30 s and the participant was asked to use the NPRS-11 to describe the severity of pain related to the occlusion in the arm. Subsequently, the cuff inflation was adjusted until the pain intensity reached 3/10 on the NPRS. The previously described TS procedure was then repeated with the cuff inflated at this lower level, with the arm relaxed. CPM was calculated by subtracting the post-conditioning PPT from the baseline PPT at the index finger region (CPM = PPT_post – PPT_pre). A positive CPM value reflects an effective endogenous inhibitory response, while lower or negative CPM values indicate reduced inhibitory capacity [17,39].

#### Psychological factors

3. To assess kinesiophobia, we used the reliable and validated version of the Fear Avoidance Beliefs Questionnaire (FABQ). This questionnaire consists of 16 statements rated on a 7-point scale (from 0 = totally agree to 6 = totally disagree) with a total score range of 0–66. Higher scores indicate higher levels of fear-avoidance beliefs [41].
4. To evaluate catastrophising in patients with CLBP, we utilised the validated Pain Catastrophizing Scale (PCS), which has shown internal consistency, test-retest reliability, and sensitivity to change. The PCS comprises 13 items rated on a 5-point scale ranging from 0 (never) to 4 (always) with a total score range of 0–52, measuring three components of catastrophising: rumination, magnification, and helplessness; higher scores indicate greater levels of pain-related catastrophising [42].
5. To evaluate anxiety, only the Anxiety subscale of the Hospital Anxiety and Depression Scale (HADS-A) was administered. This is a 7-item self-reported screening scale. Each item is scored on a 4-point Likert scale (0 = ‘as much as I always do’; 1 = ‘not quite so much’; 2 = ‘definitely not so much’; and 3 = ‘not at all’), resulting in maximum scores for anxiety of 21 points, with higher scores indicating greater levels of anxiety [43].

#### Functional outcomes

6. The lumbar flexion-evoked pain threshold was measured using a 3-Space Fastrack motion analysis system for the observational study. This system is a validated electro-goniometer for assessing lumbar mobility in patients with LBP and has demonstrated high reliability. Two motion sensors were placed on the spinous processes of T12 and S1 to monitor lumbar spine movements [44,45] The participants were then asked to bend their lumbar spine until the onset of pain.
7. Functional capacity was evaluated using the STS60 test which required individuals with CLBP to stand up and sit down on a chair without arm rests as many times as possible within 1 minute. The participants were informed when 15 seconds remained, but no encouragement was provided by the instructor during the test. The number of correct STS cycles fully completed within 1 minute was recorded for further analysis [25].

### Statistical analysis

Statistical analysis was performed using SPSS software (version 27.0, Macintosh OS, IBM Corp., Armonk, NY, USA). Normality of all variables was confirmed using the Shapiro–Wilk test, which supported the use of parametric analyses, including Pearson’s correlation coefficients. Therefore, we employed Pearson’s correlation analysis to examine the relationships between variables (pain intensity, QST parameters, psychological factors, lumbar flexion-evoked pain threshold, and functional capacity). The correlation coefficient was stratified into five levels according to the following cut-off points: a correlation coefficient of *r* = 1–0.9 was considered a very strong correlation; *r* = 0.89–0.7 indicated a strong correlation; *r* = 0.69–0.4 indicated a moderate correlation; *r* = 0.39–0.1 indicated a weak correlation; and *r* ≤ 0.09 indicated a negligible correlation [46]. *p*-values < 0.05 were considered statistically significant. Additionally, forward stepwise regression was employed to identify the biopsychosocial variables that most accurately predicted pain and kinesiophobia. The selection of variables was preceded by assessment of correlation coefficients between independent variables and pain intensity and kinesiophobia; if a significant correlation was identified for a variable, it was included in the subsequent analysis.

## Results

Fifty individuals with CLBP voluntarily participated in this observational study. Table 1 presents an overview of the participant characteristics. The results of the bivariate Pearson correlation analyses are summarised in Table 2. Significant correlations were found between pain sensitivity and PPT_LUMBAR (r = −0.509, *p* < 0.01), CPM (r = −0.336, *p* < 0.05), STS60 (r = −0.555, *p* < 0.01), kinesiophobia (*r* = 0.341, *p* < 0.05), catastrophising (*r* = 0.545, *p* < 0.01), and anxiety (r = −0.327, *p* < 0.05). These associations indicate that higher pain levels are related to increased psychological distress and central sensitisation features, and to reduced functional performance. A complete correlation matrix is presented in Table 2. A conceptual diagram as Appendix 1, visually summarises the main associations. Stepwise multiple regression analysis in participants with CLBP indicated that PPT_lumbar was a significant and independent predictor of lumbar pain (AdjR^2^ = 0.325, *β* = −0.586, *p* < 0.001; model 1), accounting for 32.5% of the variation in lumbar pain (Table 3). In model 2, the inclusion of STS60 alongside PPT_lumbar increased the explained variation to 39.7%. Additionally, catastrophising was a significant and independent predictor of kinesiophobia (AdjR^2^ = 0.206, *β* = 0.474, *p* < 0.001), accounting for 20.6% of the variation in kinesiophobia (model 1). The inclusion of STS60 in model 2 increased the explained variation to 29.1%.

**Table 1.** Overview of the participant characteristics.

*N*	50
Age (years)	52 (± 13)
Sex (female/male percentage)	56/44
Height (cm)	169 (± 9)
Weight (kg)	75 (± 15)
Body mass index (kg/m^2^)	25.9 (± 4.1)
Pain	5 (± 1.6)
PPT_LUMBAR	5.4 (± 2.3)
TS	5.4 (± 2.7)
CPM	0.36 (± 1.40)
Kinesiophobia	52.7 (± 29.9)
Catastrophising	33 (± 11.1)
Anxiety	16.9 (± 8.9)
LFEPT	99 (± 16.1)
STS60	18 (± 6.8)

Values are expressed as the mean ± the standard deviation. N: number of participants; PPT_LUMBAR: lumbar pain pressure threshold; TS: temporal summation; CPM: conditioned pain modulation; LFEPT: lumbar flexion-evoked pain threshold; STS60: 1-Minute Sit-to-Stand test.

**Table 2.** Tabulation of Pearson correlation coefficients, pain, QST parameters, lumbar movement-evoked pain, disability, and psychological factors.

		NPRS-11	PPT_LUMBAR	TS	CPM	Kinesiophobia	Catastrophising	Anxiety	LFEPT	STS60
Pain	NPRS-11	1	**−0.509** * *	0.024	**−0.336** *	**0.341** *	**0.545** * *	**−0.327** *	**0.337** *	**−0.555** * *
Pain processing mechanisms	PPT_LUMBAR		1	−0.054	**0.270** *	**−**0.205	**−0.534** * *	**0.421** *	**−0.431** * *	**0.325** *
	TS			1	0.223	0.009	−0.095	**−**0.178	**−**0.167	0.055
	CPM				1	−0.044	−0.154	**0.316** *	**−**0.004	**0.279** *
Psychological factors	Kinesiophobia					1	**0.474** * *	**0.402** *	**−0.378** *	**−0.473** * *
	Catastrophising						1	**0.769** * *	**−0.327** *	**−0.345** *
	Anxiety							1	0.229	**−0.351** *
Functional outcomes	LFEPT								1	**0.353** *
	STS60									1

*Correlations considered significant at the 0.05 level (bilateral). ** Correlations considered significant at the 0.01 level (bilateral). NPRS-11: Pain Numerical Rating Scale; PPT_LUMBAR: lumbar pain pressure threshold; TS: temporal summation; CPM: conditioned pain modulation; LFEPT: lumbar flexion-evoked pain threshold; STS60: 1-Minute Sit-to-Stand test. Note: PPT at the index finger was used to calculate TS and CPM values and was not analysed independently in relation to clinical outcomes.

**Table 3.** Multiple stepwise linear regression analyses in lumbar pain and kinesiophobia as dependent variables.

Independent variables	*R* ^2^	Adjusted *R*^2^	*R*^2^ change	Standardised β coefficient	Collinearity statistics	
					Tolerance	VIF
Lumbar Pain						
Model 1	.343	.325	.343		1.000	1.000
PPT_Lumbar				–.586**		
Model 2	.429	.397	.086			
PPT_Lumbar				–.581**	.781	1.280
STS60				–.288**	.781	1.280
Kinesiophobia						
Model 1	.224	.206	.224		1.000	1.000
Catastrophizing				.313**		
Model 2	.323	.291	.099			
Catastrophizing				.358**	.881	1.135
STS60				–.358**	.881	1.135

PPT_LUMBAR: Pain Pressure Threshold in Lumbar; STS60: Sit to Stand 60 s; VIF: variance inflation factor.

## Discussion

To the best of our knowledge, this is the first study to investigate, from a holistic perspective, the correlations between pain intensity, pain processing mechanisms in conjunction with various psychosocial factors, and functional outcomes in patients with CLBP. Our exploratory findings revealed associations between pain intensity and various aspects of central pain processing, psychological factors, and functional outcomes in patients with CLBP.

This current study demonstrated significant correlations between pain intensity and several variables, including PPT_lumbar, CPM, lumbar flexion-evoked pain threshold, kinesiophobia, catastrophising, anxiety, and STS60 scores. These preliminary results support the relevance of a biopsychosocial framework in understanding CLBP, suggesting a complex interplay of physical, physiological, and psychological factors. Until now, no study had comprehensively examined these correlations in their entirety within a specific population with CLBP. Rather, previous research had only investigated these factors separately, except for movement-evoked pain, which to date, has only been explored in patients with chronic knee pain.

### Pain sensitivity and modulation (QST measures)

Similar to our findings, previous studies have identified correlations between pain sensitivity, PPT_lumbar, and CPM [47–50]. The significant negative correlation between pain and PPT_lumbar suggests that individuals with higher lumbar pain sensitivity (lower thresholds for reporting pain in response to pressure) tend to experience more intense pain and are more likely to report more generalised and bothersome clinical pain.^48^ Moreover, our analysis identified PPT_lumbar as a significant and independent predictor of lumbar pain in individuals with CLBP. In this sense, our model explained 32.5% of the variance in lumbar pain. This finding aligns with previous studies which have demonstrated the relevance of PPT in predicting pain sensitivity and chronic pain outcomes in musculoskeletal conditions [16 However, while reduced lumbar PPTs may reflect increased mechanical sensitivity at the local site of pain, they do not provide evidence of central sensitisation. In contrast, TS and CPM, particularly the significant association between reduced CPM and greater pain severity, offer stronger support for altered central pain modulation processes in this sample. In patients with CLBP, this hyperalgesic response is believed to reflect dysfunction in pain modulatory systems, leading to persistent pain despite the absence of significant peripheral injury [14]. CS has been shown to influence both the experience and sensitivity of pain, thereby making PPT_Lumbar a valuable predictor in clinical assessments [51]. Indeed, incorporating PPT assessments in clinical settings may enhance the identification of patients at risk of more severe pain presentations, ultimately facilitating targeted interventions aimed at modulating central sensitisation.

In turn, the significant negative correlation we found between pain and CPM implies that reduced descending inhibitory pathways may contribute to increased pain perception, as shown in previous studies demonstrating differences in CPM between individuals with and without chronic pain [18,20,21]. However, these results do not align with those presented by Teixeira et al. (2023) [23] and LeResche et al. (2013) [48]. These differences may be partly explained by the small sample size examined by Teixeira et al. or the differing protocols used in the work by LeResche et al. (thermal rather than tactile stimuli). Therefore, our results are consistent with the notion that CS plays a crucial role in CLBP, [11] although additional work will be needed to assess the effects of clinical pain on CPM.

Interestingly, our findings revealed that TS did not significantly correlate with pain sensitivity. As in previous studies, this lack of meaningful correlation might suggest that TS, as a measure of CS, operates independently from other pain-related factors such as pain intensity, psychological factors, and functional outcomes [52]. Although TS and CPM are regarded as reflective of nociceptive signal processing within the CNS, they operate in distinct areas and can influence or be influenced by different variables. TS occurs at the level of dorsal horn neurons, where it interacts with incoming afferent signals, whereas CPM is mediated at the brainstem level and interacts with outcoming efferent signals [52]. Further research will be necessary to clarify the precise mechanisms underpinning these findings and to explore whether specific subgroups of patients with CLBP exhibit distinct patterns of TS [52].

### Psychosocial factors and pain

We found associations between pain and kinesiophobia, in line with previous studies that have similarly identified significant correlations between pain intensity and psychosocial factors such as kinesiophobia, [53] catastrophising, [54] and anxiety [55]. This suggests that individuals with CLBP who experience higher levels of kinesiophobia, catastrophising, and anxiety tend to experience more intense pain. These positive correlations underscore the impact of cognitive and emotional factors on pain experience, as explained in the Fear-Avoidance Model [7–9]. Moreover, the results of our study indicated that catastrophising is a significant predictor of kinesiophobia, accounting for 20.6% of its variation, which also corresponds with previous work [56].

Finally, catastrophising, characterised by an exaggerated negative mindset towards pain, exacerbates fear-avoidance behaviours, leading to greater kinesiophobia and functional limitations [7–9,57]. Importantly, our study extended this relationship by incorporating the STS60, thereby demonstrating that physical deconditioning significantly adds to the explained variation in kinesiophobia, increasing it to 29.1%. This suggests that decreased functional capacity, measured by the STS60, not only correlates with and predicts pain but also contributes to fear-avoidance behaviours (kinesiophobia), because reduced functional capacity likely amplifies concerns about potential harm during movement. The association between functional performance and kinesiophobia has been widely documented in other contexts, although specific studies focusing on the STS60 as a predictor of kinesiophobia are still limited.

### Functional capacity and clinical pain outcomes

Moreover, our findings further indicated a negative correlation between lumbar pain and STS60 performance. Indeed, the inclusion of the STS60 alongside PPT_lumbar in the regression model significantly increased the explained variance in lumbar pain to 39.7%. This finding line up with previous research suggesting a negative association between functional capacity and pain intensity in chronic musculoskeletal conditions [58]. Specifically, it supports the notion that individuals with increased levels of pain experience a reduced capacity for functional activities [59]. In this context, poorer performance in the STS60 test may reflect reduced functional capacity and lower extremity endurance, both of which are crucial for maintaining functional independence, [60] although further research is needed to clarify this relationship.

This aforementioned association also highlights the utility of the STS60 as a quick, simple and clinically meaningful metric for functional capacity in individuals with CLBP. Furthermore, the Fear-Avoidance Model highlights how reduced physical function may increase fear-avoidance behaviours, leading to further deconditioning and worsening pain perception over time [7–9]. Hence, this negative cycle between pain, reduced functional capacity, and avoidance of movement makes the STS60 a relevant and practical means to assess functional limitations and predict pain outcomes in patients with CLBP.

Moreover, the significant negative correlation between pain and the flexion-evoked pain threshold identified in this current work indicates that lower thresholds for pain during lumbar flexion are associated with higher levels of overall pain. This finding aligns with previous research in knee osteoarthritis [59] and suggests that individuals with CLBP who experience greater pain during lumbar flexion may have heightened sensitivity and reduced tolerance to movement-related pain, also reflecting the involvement of CS [59]. This concurs with previous research indicating that movement-evoked pain thresholds can be indicative of central pain mechanisms (i.e. CS) which can influence several chronic pain conditions including CLBP [59,61].

### Exploratory relationships between QST and function

Our results showed significant correlations between PPT and several key factors in patients with CLBP, including CPM, flexion-evoked pain threshold, and STS60 scores. Firstly, the significant correlation between PPT and CPM underscores the intricate balance between pain sensitivity and descending inhibitory pathways in patients with CLBP. This finding aligns with previous research indicating that individuals with reduced pain thresholds often exhibit low endogenous pain inhibition, suggesting a potential dysfunction in the central processing of nociceptive signals (CS) [59,62]. Moreover, the correlation between PPT and flexion-evoked pain threshold highlights the relationship between mechanical pain sensitivity and movement-evoked pain threshold. This suggests that patients with lower PPTs are likely to experience a lower lumbar movement-evoked pain threshold, possibly because of CS or somatosensory cortical reorganisation. Nonetheless, this new association still requires further investigation.

The positive correlation between PPT and functional capacity (STS60), indicates that increased pain sensitivity is associated with greater functional impairment. This is consistent with the notion that heightened pain perception can limit physical activity and daily functioning. Similar findings in previous studies support this relationship, suggesting that interventions aimed at reducing pain sensitivity could potentially improve functional outcomes in individuals with CLBP [59,63]. This relationship may be explained by fear-avoidance behaviour and physical deconditioning. Thus, patients with higher pain sensitivity might be less inclined to engage in physical activities, leading to reduced functional capacity and further perpetuating the pain cycle [64]. Given that both factors are predictors of pain intensity, they should be considered as target objectives for assessment and treatment strategies in cases of CLBP.

Finally, our analysis indicated that CPM showed significant correlations with both the flexion-evoked pain threshold and STS60 score. These findings concur with a previous study and suggest that the efficiency of descending pain inhibitory pathways, as measured by CPM, is linked to higher thresholds for pain during lumbar flexion and with better functional capacity [59]. This correlation may imply that individuals with more effective endogenous pain modulation experience less pain during lumbar movements and exhibit better overall physical performance.

It is worth mentioning that this observational study may be susceptible to selection bias. The correlational analyses presented are exploratory in nature and serve to identify preliminary associations without adjustment for confounders. These results should be interpreted with caution and are intended to guide future confirmatory research using multivariable modelling. Furthermore, the design of this work limits the ability to infer causality. Therefore, future research should involve larger, randomised, longitudinal studies to better understand causal relationships and explore the effectiveness of interventions aimed at modulating pain sensitivity and improving pain modulatory mechanisms in patients with CLBP. Moreover, the exclusion of participants over 65 years may limit the generalisability of our findings to older populations with CLBP. Finally, given the exploratory nature of this study, we focused on identifying potential associations using a feasible sample size, which still provided adequate power to detect medium effect sizes. These findings should be interpreted with caution and considered preliminary until validated in larger samples.

Nevertheless, this study is noteworthy because it is the first to investigate, from a holistic perspective, the correlations between pain intensity, pain processing mechanisms, and various psychosocial factors in individuals with CLBP. Pain intensity and kinesiophobia were associated with various central processing mechanisms of chronic pain (QST), psychological factors, and functional outcomes in individuals with CLBP, and could be predicted by lumbar PPT, STS60 performance, and catastrophising. Moreover, the identification of significant variables in our regression analysis provides evidence for their roles in chronic pain. Furthermore, the comprehensive analysis of the interplay between biophysical, psychological, and functional factors underscores the multifaceted nature of CLBP, which may inform more integrated and effective assessments and intervention strategies in cases of CLBP in the future. Additionally, incorporating PPT_Lumbar and STS60 assessments into clinical settings may enhance the identification of patients at risk of more severe pain and kinesiophobia presentations, ultimately facilitating targeted interventions aimed at modulating CS.

In summary, these correlations highlight the intricate interplay between pain sensitivity, pain modulatory mechanisms, psychological factors, and functional outcomes in individuals with CLBP. These exploratory results underscore the complex and multifactorial nature of chronic pain and suggest that future research should further investigate these relationships to inform comprehensive assessment and treatment approaches. Moreover, new functional and physiological predictors of pain and kinesiophobia may contribute to the development of better assessment and treatment protocols for CLBP. From a clinical perspective, lumbar PPT assessments and the STS60 test could serve as accessible, time-efficient screening tools to inform individualised treatment planning. For instance, lower PPTs may indicate a need for interventions targeting altered pain processing, such as graded exposure or sensory desensitisation strategies, whereas reduced STS60 performance may suggest the importance of physical reconditioning and exercise-based approaches [65]. Integrating these measures into multidisciplinary assessment models may enhance early risk identification and improve outcomes in CLBP management. Future research should further therefore investigate these relationships and continue to explore interventions targeting both pain neurophysiology and psychological factors to improve outcomes in patients with CLBP and to understand the mechanisms behind these improvements.

## Data Availability

The authors confirm that the data supporting the findings of this study are available within the article or on request from the corresponding author.
